# Liraglutide, a GLP-1 Receptor Agonist, Which Decreases Hypothalamic 5-HT2A Receptor Expression, Reduces Appetite and Body Weight Independently of Serotonin Synthesis in Mice

**DOI:** 10.1155/2018/6482958

**Published:** 2018-02-01

**Authors:** Katsunori Nonogaki, Takao Kaji

**Affiliations:** Department of Diabetes Technology, Tohoku University Graduate School of Biomedical Engineering, Sendai, Japan

## Abstract

A recent report suggested that brain-derived serotonin (5-HT) is critical for maintaining weight loss induced by glucagon-like peptide-1 (GLP-1) receptor activation in rats and that 5-HT2A receptors mediate the feeding suppression and weight loss induced by GLP-1 receptor activation. Here, we show that changes in daily food intake and body weight induced by intraperitoneal administration of liraglutide, a GLP-1 receptor agonist, over 4 days did not differ between mice treated with the tryptophan hydroxylase (Tph) inhibitor p-chlorophenylalanine (PCPA) for 3 days and mice without PCPA treatment. Treatment with PCPA did not affect hypothalamic 5-HT2A receptor expression. Despite the anorexic effect of liraglutide disappearing after the first day of treatment, the body weight loss induced by liraglutide persisted for 4 days in mice treated with or without PCPA. Intraperitoneal administration of liraglutide significantly decreased the gene expression of hypothalamic 5-HT2A receptors 1 h after injection. Moreover, the acute anorexic effects of liraglutide were blunted in mice treated with the high-affinity 5-HT2A agonist (4-bromo-3,6-dimethoxybenzocyclobuten-1-yl) methylamine hydrobromide 14 h or 24 h before liraglutide injection. These findings suggest that liraglutide reduces appetite and body weight independently of 5-HT synthesis in mice, whereas GLP-1 receptor activation downregulates the gene expression of hypothalamic 5-HT2A receptors.

## 1. Introduction

Central serotonin (5-HT) systems have a critical role in the regulation of appetite and body weight. Central serotonin 5-HT2C receptors contribute to the leptin-independent regulation of appetite [1]. Liraglutide, a glucagon-like peptide-1 (GLP-1) receptor agonist used clinically to treat type 2 diabetes and/or obesity, induces feeding suppression in mice 1 h after administration [2]. We previously reported that the acute anorexic effects of liraglutide in mice do not require central 5-HT and functional leptin receptor signaling [2]. A recent report by Anderberg et al., however, suggested that brain-derived 5-HT is critical for maintaining weight loss induced by GLP-1 receptor activation and pharmacologic blockade of central serotonin 5-HT2A receptors using R-96544 attenuates the chronic anorexic and weight loss effects of central injection of exedin-4 (EX4) or intraperitoneal injection of liraglutide in rats [3].

Therefore, in the present study, to determine whether 5-HT is critical for the weight loss induced by GLP-1 receptor activation in mice, we examined the effects of liraglutide on daily food intake and body weight over 4 days in mice treated with or without the tryptophan hydroxylase inhibitor p-chlorophenylalanine (PCPA) for 3 days. In addition, to evaluate the relationship between the acute anorexic effects of the GLP-1 receptor agonist and the expression of hypothalamic 5-HT2A receptors, we examined the effect of liraglutide on the expression of hypothalamic 5-HT2A and 5-HT2C receptors in mice, which are responsive to the administration of liraglutide. We further assessed whether 5-HT2A receptors are involved in the acute anorexic effects of liraglutide by examining the acute effects of liraglutide on food intake in mice pretreated with the high-affinity 5-HT2A agonist (4-bromo-3,6-dimethoxybenzocyclobuten-1-yl) methylamine hydrobromide (TCB-2).

## 2. Materials and Methods

### 2.1. General Procedures

Male C57BL6J mice were purchased from Japan CLEA. The mice were individually housed in cages with free access to water and chow pellets in a light- and temperature-controlled environment (12 h on/12 h off, lights on at 08 : 00; 20–22°C).

In exp 1, 6-week-old C57BL6J mice were intraperitoneally injected with 1% Tween saline or PCPA (500 mg/kg) once a day over 3 days as described previously [2]. Daily body weight changes were determined. In the fourth day, the animals were decapitated and the hypothalamus was removed for RNA extraction, as described previously [2, 4].

In exp 2, 6-week-old C57BL6J mice were intraperitoneally injected with 1% Tween saline or PCPA (500 mg/kg) once a day over 3 days. Then, mice were intraperitoneally injected with saline or liraglutide (100 *μ*g/kg) in the light cycle once a day over 4 days. Daily food intake and body weight changes were determined.

In exp 3, 6-week-old C57BL6J mice were intraperitoneally injected with saline or liraglutide (100 *μ*g/kg) in the light cycle. One hour later, the animals were decapitated; animals were not fed. The hypothalamus was removed for RNA extraction, as described previously [2, 4].

In exp 4, 6-week-old C57BL6J mice were intraperitoneally injected with saline or the high-affinity 5-HT2A agonist, 4-Bromo-3,6-dimethoxybenzocyclobuten-1-yl methylamine hydrobromide (TCB-2) (2.5 mg/kg) in the light cycle. 14 h later and 24 h later, mice were intraperitoneally injected with saline or liraglutide (100 *μ*g/kg) in the light cycle, respectively. Chow pellets were provided 30 min later. The intake of chow pellets was measured for the next 1 h and then 2 h.

The dose of PCPA (500 mg/kg) was selected based on evidence that PCPA remarkably decreases brain and serum 5-HT levels in mice [5, 6]. The dose of liraglutide (100 *μ*g/kg) was selected based on evidence that liraglutide acutely induced hypophagia in mice [2, 7]. The dose of TCB-2 (2.5 mg/kg) was selected based on evidence that TCB-2 had no significant effect on food intake [8].

Liraglutide was a kind gift from Novo Nordisk, Japan. Sarpogrelate hydrochloride was supplied by Mitsubishi Pharma (Osaka, Japan). TCB-2 was purchased from Tocris Bioscience (Ellisville, MO, USA).

Liraglutide was dissolved in 0.2 ml 0.9% saline. The PCPA was suspended in 0.2 ml 1% Tween saline. The experiment was performed between 9 : 00 and 12 : 00. The animal studies were conducted in accordance with the institutional guidelines for animal experiments at the Tohoku University Graduate School of Medicine.

### 2.2. Real-Time Quantitative Reverse Transcription-Polymerase Chain Reaction (RT-PCR)

Total RNA was isolated from mouse hypothalamus using the RNeasy Midi kit (Qiagen, Hilden, Germany) according to the manufacturer's instructions. cDNA synthesis was performed using a Super Script III First-Strand Synthesis System for RT-PCR Kit (Invitrogen, Rockville, MD) with 1 *μ*g total RNA. cDNA synthesized from total RNA was evaluated in a real-time PCR quantitative system (LightCycler Nano Instrument Roche Diagnostics, Mannheim, Germany). The primers used were listed in Table 1.

The relative amount of mRNA was calculated using *β*-actin mRNA as the invariant control. Data are shown as fold change of the mean value of the control group, which received saline as described previously [2, 4].

#### 2.2.1. Statistical Methods

Data are presented as mean ± SEM (*n* = 6). Comparisons between the two groups were performed using Student's *t*-test. Comparisons among more than two groups were performed using analysis of variance with Bonferroni's correction for multiple comparisons. A *P* value of less than 0.05 was considered statistically significant.

## 3. Results

### 3.1. Effects of PCPA for 3 Days on Food Intake, Body Weight Changes, and Expression of Hypothalamic Tph2 and 5-HT2AR in Mice following Treatment with or without PCPA for 3 Days

**Figure 1:**
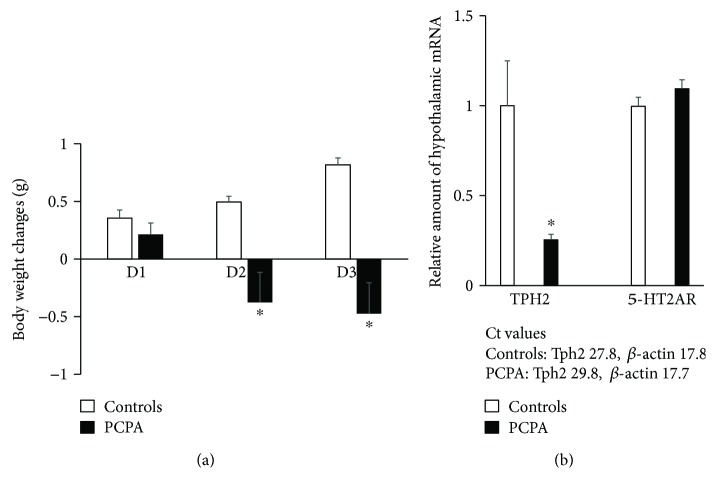
Effects of intraperitoneal injection of PCPA (500 mg/kg) for 3 days on body weight changes (a) and expression of hypothalamic Tph2 and 5-HT2A receptors (b) in mice. Basal body weight was 22.1 ± 0.2 g in controls and 21.8 ± 0.2 g in the PCPA-treated mice. Data are presented as the mean values ± SEM (*n* = 6/group of animals). ^∗^*P* < 0.05.

Treatment with PCPA (500 mg/kg) for 3 days significantly decreased body weight gain (Figure 1(a)) compared with controls. Treatment with PCPA for 3 days remarkably decreased expression of hypothalamic Tph2 compared with controls (25%) but did not affect the expression of hypothalamic 5-HT2A receptors in mice (Figure 1(b)). These findings demonstrate that despite the inhibition of Tph2, the treatment with PCPA does not affect expression of hypothalamic 5-HT2A receptors in mice.

### 3.2. Effects of Liraglutide on Daily Food Intake and Body Weight Changes in Mice following Treatment with or without PCPA for 3 Days

**Figure 2:**
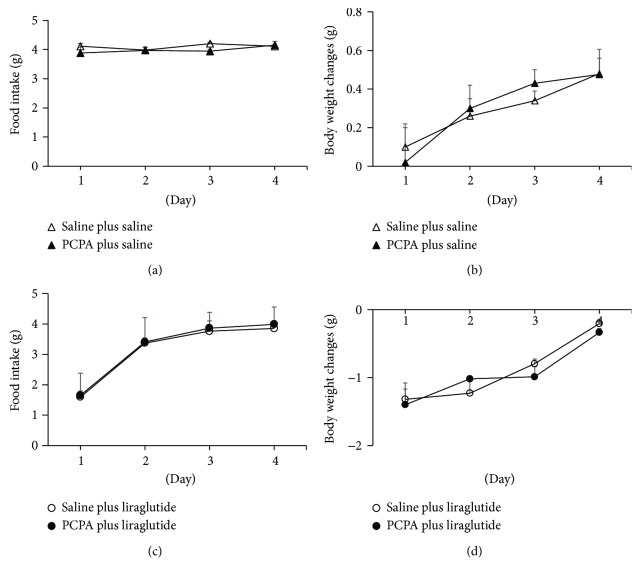
Effects of intraperitoneal injection of saline over 4 days on daily food intake (a) and body weight changes (b) in mice treated with or without PCPA (500 mg/kg) for 3 days. Effects of intraperitoneal injection of liraglutide (100 *μ*g/kg) over 4 days on daily food intake (c) and body weight changes (d) in mice treated with or without PCPA (500 mg/kg) for 3 days. Basal body weight of mice without PCPA was 22.3 ± 0.2 g, and basal body weight of mice treated with PCPA was 20.3 ± 0.6 g. Open triangle: mice treated with saline following treatment with 1% Tween saline alone for 3 days; closed triangle: mice treated with saline following treatment with PCPA for 3 days; open circle: mice treated with liraglutide following treatment with 1% Tween saline alone for 3 days; filled circle: mice treated with liraglutide following treatment with PCPA for 3 days. Data are presented as the mean values ± SEM (*n* = 6/group of animals).

Changes in daily food intake (Figure 2(a)) and body weight (Figure 2(b)) induced by intraperitoneal administration of saline over 4 days did not differ between mice treated with the PCPA (500 mg/kg) for 3 days and mice without PCPA treatment. Intraperitoneal administration of liraglutide (100 *μ*g/kg) in mice significantly decreased food intake on the first day of treatment and decreased body weight over 4 days compared with saline controls in mice treated with or without PCPA for 3 days. Changes in daily food intake (Figure 2(c)) and body weight (Figure 2(d)) induced by intraperitoneal administration of liraglutide (100 *μ*g/kg) over 4 days did not differ between mice treated with the PCPA (500 mg/kg) for 3 days and mice without PCPA treatment. These findings demonstrate that 5-HT is not required for feeding suppression and body weight loss induced by liraglutide in mice.

### 3.3. Effect of Liraglutide on Expression of Hypothalamic 5-HT2A Receptors and 5-HT2C Receptors in Mice

**Figure 3:**
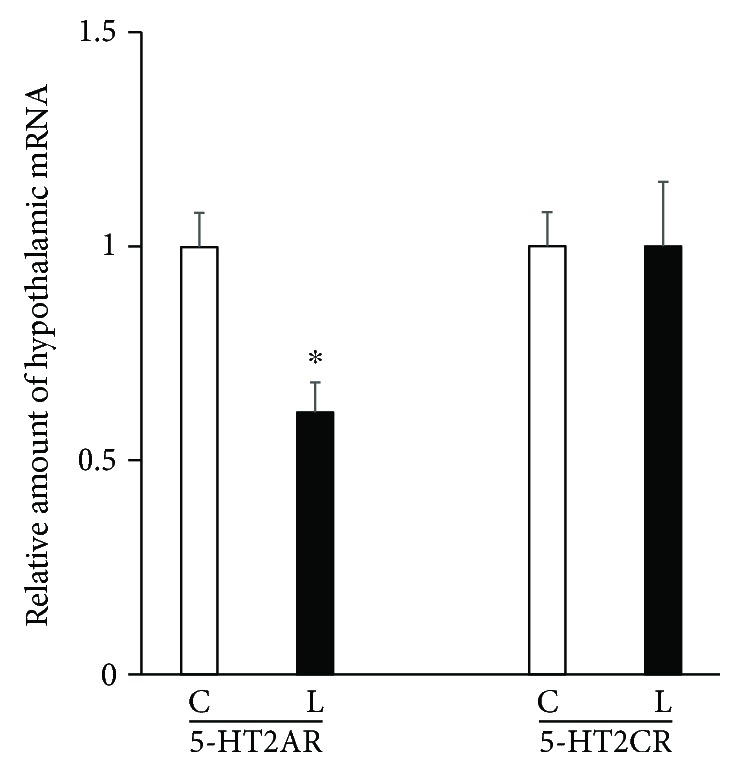
Effects of intraperitoneal injection of liraglutide (100 *μ*g/kg) or saline on the expression of hypothalamic 5-HT2A receptors (5-HT2AR) and 5-HT2C receptors (5-HT2CR) in C57BL6J mice. C, saline controls; L, liraglutide-treated mice. Data are presented as the mean values ± SEM (*n* = 6/group of animals). ^∗^*P* < 0.05.

Intraperitoneal injection of liraglutide (100 *μ*g/kg) significantly decreased the expression of hypothalamic 5-HT2A receptors compared with saline controls but did not affect the expression of hypothalamic 5-HT2C receptors 1 h after injection (Figure 3). These findings demonstrate that GLP-1 receptors downregulate expression of hypothalamic 5-HT2A receptors in mice.

### 3.4. Effect of Pretreatment with TCB-2 on the Acute Anorexic Effect of Liraglutide in Mice

**Figure 4:**
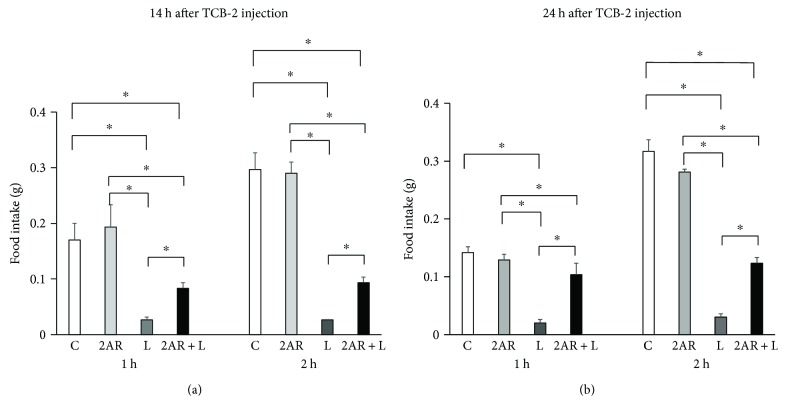
Effects of intraperitoneal injection of liraglutide (100 *μ*g/kg) or saline on food intake in C57BL6J mice intraperitoneally injected with TCB-2 (2.5 mg/kg). Liraglutide was injected 14 h later (a) or 24 h later (b). Intake of chow pellets was measured for the next hour and then at 2 h. Body weight of C57BL6J mice without TCB-2 was 23.9 ± 0.2 g in the saline controls and 24.2 ± 0.3 g in the liraglutide-treated group. Body weight of C57BL6J mice treated with TCB-2 was 23.9 ± 0.2 g in the saline controls and 24.2 ± 0.3 g in the TCB-2 and liraglutide-treated group. C, saline controls; 2AR, TCB-2L treated mice; L, liraglutide-treated mice, and 2AR + L, TCB-2 and liraglutide-treated mice. Data are presented as the mean values ± SEM (*n* = 6/group of animals). ^∗^*P* < 0.05.

Although intraperitoneal injection of liraglutide (100 *μ*g/kg) significantly suppressed food intake compared with saline controls for 2 h after injection, the acute anorexic effects of liraglutide were blunted in mice, which were pretreated with TCB-2 (Figures 4(a) and 4(b)). These findings demonstrate that pretreatment with a 5-HT2A agonist partially reverses the acute anorexic effect of liraglutide in mice.

## 4. Discussion

The present study demonstrated that PCPA treatment (500 mg/kg) for 3 days induces body weight loss in mice. These findings support a previous report by other investigators that treatment with PCPA (300 mg/kg) suppresses weight gain by increasing energy expenditure [9].

Although Tph1-deficient mice fed a normal diet have no significant effect on body weight, Tph1-deficient mice fed a high-fat diet are protected from obesity and insulin resistance by promoting brown adipose tissue-mediated thermogenesis [10]. On the other hand, Tph2-deficient mice fed a normal diet decrease food intake and body weight [11]. Because PCPA inhibits Tph1 and Tph2, both mechanisms could contribute to weight loss.

Although liraglutide reportedly increases brain 5-HT levels in rats [3] and both liraglutide and serotonergic receptor agonists, like lorcaserin, reduced body weight and food intake in rats, the increase in brain 5-HT levels does not always induce reductions of food intake and body weight, especially in mice. Despite increased brain 5-HT levels, ob/ob mice display hyperphagia and obesity [12]. The genetic inhibition of 5-HT synthesis in the brainstem decreases food intake and body weight in ob/ob mice and the wild-type mice [12]. In addition, the pharmacologic inhibition of 5-HT synthesis in the brainstem induced by treatment with a high dose PCPA (500 mg/kg) or trans-2 PCPA decreases body weight and food intake in C57BL6J mice, db/db mice [2], and high-fat diet-induced obesity [13]. Thus, the decrease in central 5-HT leads to the decrease in body weight and food intake in mice.

We previously reported that the pharmacologic inhibition of 5-HT synthesis does not affect the acute anorexic effects of liraglutide in mice [2]. The results of the present study further demonstrated that the pharmacologic inhibition of 5-HT synthesis did not affect the feeding suppression induced by liraglutide for 24 h after injection in mice. Moreover, the inhibition of 5-HT synthesis did not affect both the acute weight loss and maintenance of weight loss induced by liraglutide in mice. Thus, the feeding suppression and weight loss induced by liraglutide could be independent of 5-HT synthesis in mice.

In addition, we demonstrated that liraglutide acutely decreased the gene expression of hypothalamic 5-HT2A receptors in mice, which are responsive to the acute anorexic effects of liraglutide. Despite the chemical inhibition of 5-HT synthesis, the gene expression of hypothalamic 5-HT2A receptors was not changed. Thus, changes in brain 5-HT synthesis and the gene expression of hypothalamic 5-HT2A receptors could be independent, and the decrease in hypothalamic 5-HT2A receptor gene expression induced by liraglutide is unlikely to be compensatory to an increased brain 5-HT levels, although protein levels of the receptor were not measured here and could represent another regulatory mechanism.

Moreover, pretreatment with a 5-HT2A agonist reduced the acute anorexic effects of liraglutide. We previously reported that intraperitoneal injection of sarpogrelate hydrochloride, a blood-brain barrier-penetrating selective 5-HT2A receptor antagonist, acutely suppresses food intake and chronic administration of sarpogrelate hydrochloride decreases daily food intake and body weight in mice [14]. These results suggest that GLP-1 receptors downregulate the expression of 5-HT2A receptors in the hypothalamus and decreased hypothalamic 5-HT2A receptor signaling might be involved in the acute anorexic effects of GLP-1 receptor agonists independently of brain 5-HT.

We cannot rule out that pretreatment with a 5-HT2A agonist might temporarily reduce the downstream signal of a 5-HT2A pathway; thus, the particular timing employed here has a potential to work as an antagonizing treatment to the 5-HT2A signaling pathway. Because the pretreatment might reduce the amount of functional 5-HT2A receptors at the downstream signaling, liraglutide might not be able to induce a full anorexic effect. In addition, the similar effects of 5-HT2A receptor agonist and antagonist on food intake [14–16] might be due to the paradoxical phenomenon that both agonism and antagonism of 5-HT2A receptors induce 5-HT2A receptor's desensitization or downregulation [17].

These results are in complete contrast to recently reported findings by Anderberg et al. that brainstem-derived 5-HT is critical for maintaining the weight loss induced by GLP-1 receptor activation and that central blockade of 5-HT2A receptors attenuates the weight loss and anorexic effects of GLP-1 receptor agonists in rats [3]. Although the dose of PCPA (500 mg/kg) that we used was higher than that (100 mg/kg) used in their study, the PCPA treatment methods were the same between their study and ours [3]. The different results might be due to species-specific differences between rats and mice or the paradoxical phenomenon of 5-HT2A receptor agonists and antagonists [17].

Interestingly, effect of liraglutide on food intake in mice is only eliciting an anorexic effect on the first day of treatment. This is not the case for rats, which show a multiday anorexic response that is the primary driver of the weight loss [3]. Despite the anorexic effect of liraglutide disappearing after the first day of treatment in mice, the body weight loss induced by liraglutide persisted for 4 days. These findings suggest that increased energy expenditure is involved in maintaining weight loss induced by liraglutide in mice. Liraglutide reportedly stimulates the central nervous system-mediated brown adipose tissue thermogenesis and adipocyte browning independent of food intake in mice [18]. Because this profile and potential mechanisms of weight loss induced by liraglutide are different between rats in the Anderberg study [3] and mice in the present study, it is not at all surprising that different brain factors are engaged by liraglutide in these two species. In addition, the effect of 5-HT2A agonist on feeding seems to be opposite between mice and rats, at least by comparing the studies. Moreover, our study demonstrated that liraglutide acutely decreased the expression of hypothalamic 5-HT2A receptors and food intake in mice.

## 5. Conclusions

These findings suggest that liraglutide reduces appetite and body weight independently of 5-HT synthesis and that GLP-1 receptors downregulate the expression of hypothalamic 5-HT2A receptors in mice. Pretreatment with a 5-HT2A agonist might suppress the acute anorexic effects of liraglutide in mice.

## Figures and Tables

**Table 1:** Primers used for real-time RT-PCR.

Gene	Primer	Sequence
5-HT2AR	Sense	TTC AGT GCC AGT ACA AGG AG
	Antisense	GAG TGT TGG TTC CCT AGT GTA A
5-HT2CR	Sense	CTG AGG GAC GAA AGC AAA G
	Antisense	CAC ATA GCC AAT CCA AAC AAA C
Tph2	Sense	CGT GTG TGA AAT CCT TTG AC
	Antisense	GGG GTT GAA GTA TAC CGA GA
*β*-Actin	Sense	TTG TAA CCA ACT GGG ACG ATA TGG
	Antisense	GAT CTT GAT CTT CAT GGT GCT AGG

5-HT2AR: 5-HT2A receptor; 5-HT2CR: 5-HT2C receptor.
